# Myo1f, an Unconventional Long-Tailed Myosin, Is a New Partner for the Adaptor 3BP2 Involved in Mast Cell Migration

**DOI:** 10.3389/fimmu.2019.01058

**Published:** 2019-05-09

**Authors:** Arnau Navinés-Ferrer, Erola Ainsua-Enrich, Eva Serrano-Candelas, Joan Sayós, Margarita Martin

**Affiliations:** ^1^Biochemistry Unit, Biomedicine Department, Faculty of Medicine, University of Barcelona, Barcelona, Spain; ^2^Laboratory of Clinic and Experimental Immunoallergy, IDIBAPS, Barcelona, Spain; ^3^Immune Regulation and Immunotherapy Group, CIBBIM-Nanomedicine, Vall d'Hebron University Hospital, Research Institute (VHIR), Autonomous University of Barcelona, Barcelona, Spain

**Keywords:** adaptor molecules, unconventional myosins, KIT signaling, mast cells, cell migration and adhesion, cytoske leton

## Abstract

Mast cell chemotaxis is essential for cell recruitment to target tissues, where these cells play an important role in adaptive and innate immunity. Stem cell factor (SCF) is a major chemoattractant for mast cells. SCF binds to the KIT receptor, thereby triggering tyrosine phosphorylation in the cytoplasmic domain and resulting in docking sites for SH2 domain-containing molecules, such as Lyn and Fyn, and the subsequent activation of the small GTPases Rac that are responsible for cytoskeletal reorganization and mast cell migration. In previous works we have reported the role of 3BP2, an adaptor molecule, in mast cells. 3BP2 silencing reduces FcεRI-dependent degranulation, by targeting Lyn and Syk phosphorylation, as well as SCF-dependent cell survival. This study examines its role in SCF-dependent migration and reveals that 3BP2 silencing in human mast cell line (LAD2) impairs cell migration due to SCF and IgE. In that context we found that 3BP2 silencing decreases Rac-2 and Cdc42 GTPase activity. Furthermore, we identified Myo1f, an unconventional type-I myosin, as a new partner for 3BP2. This protein, whose functions have been described as critical for neutrophil migration, remained elusive in mast cells. Myo1f is expressed in mast cells and colocalizes with cortical actin ring. Interestingly, Myo1f-3BP2 interaction is modulated by KIT signaling. Moreover, SCF dependent adhesion and migration through fibronectin is decreased after Myo1f silencing. Furthermore, Myo1f silencing leads to downregulation of β1 and β7 integrins on the mast cell membrane. Overall, Myo1f is a new 3BP2 ligand that connects the adaptor to actin cytoskeleton and both molecules are involved in SCF dependent mast cell migration.

## Introduction

Mast cell recruitment into connective tissue in a healthy or pathological situation requires cell adhesion, spreading and migration, and these events are transduced from external stimuli to cytoskeleton rearrangements. Most established mast cell chemoattractants are antigens recognized by immunoglobulins bound to FcεRI and stem cell factor (SCF), the ligand for the KIT receptor, a type III tyrosine kinase receptor (1). KIT signal transduction is crucial for mast cell growth, survival and differentiation, as well as for the migration and homing of mast cells into target tissues. KIT through PI3 kinase signaling influences mast cell growth and survival (2). SCF is a major chemotactic attractant for mast cells and their precursors. Once bound to KIT, SCF causes KIT tyrosine phosphorylation and formation of docking sites for SH2 domain-containing molecules, such as Lyn and Fyn. Activation of Fyn leads to phosphorylation of Gab2 and subsequent activation of the small GTPase Rac that is responsible for the cytoskeletal reorganization and mast cell migration (3).

The SH3-binding protein 2 (3BP2) is a cytoplasmic adapter protein originally identified to bind to the tyrosine kinase Abl SH3 domain (4). Human 3BP2 is a 561-aa protein containing an N-terminal pleckstrin homology (PH) domain, an SH3-binding proline-rich region, and a C-terminal SH2 domain. 3BP2 positively regulates mast cell responses through FcεRI and KIT receptors (5, 6). The knockdown expression of 3BP2 reduces degranulation, cytokine secretion and mast cell survival (5, 6). It has been reported that 3BP2 is required for optimal activation of Src family kinases and small GTPase Rac2 that regulates chemoattractant-mediated neutrophil activation, and motility. Consequently, the loss of 3BP2 increases susceptibility to infections such as *Listeria monocytogenes*. These functional defects are partially explained by the failure to fully activate Vav1 (7). Vav1 is a guanosine-nucleotide-exchange factor (GEF) for members of the Rho GTPase family (8). RhoA, Rac1,2,3, and Cdc42 belong to the family of Rho GTPases that regulates a range of biological response pathways, including cell motility and actin dynamics (9).

This work aims to investigate the role of 3BP2 in cytoskeleton reorganization and mast cell migration. In order to shed light on the molecules involved in 3BP2 signalosome, the three-hybrid screening system was performed on a bone marrow expression library using human 3BP2 as bait. Vav1 and Myo1f were found to be ligand partners for 3BP2. Vav1 has previously been reported to bind to Y183 3BP2 (10), but Myo1f represents a novel binding ligand for 3BP2 and its expression and function in mast cells remain elusive.

Myo1f is a long-tailed unconventional class I myosin whose gene is located on the chromosome 19 (19p13.3-p13.2) (11). Myosins are actin-dependent molecular motors that use the energy from ATP hydrolysis to move along actin filaments (12). Class I myosins are evolutionarily ancient, represent the largest group of unconventional myosins and exist in a wide range of species. Mice and humans have a total of eight class I myosin heavy-chain genes, six of which encode short-tailed forms (Myo1a, b, c, d, g and h) and two of which encode long-tailed (amoeboid) forms (Myo1e and f). All class I myosins consist of an N-terminal motor domain, light-chain-binding IQ motifs (calmodulin binding) and a basic tail homology 1 (TH1) domain thought to affect interactions with membranes. Long-tailed class I myosins have an additional proline-rich TH2 domain and a TH3 domain containing a single Src homology 3 (SH3) domain (11). The Myo1f transcript has been described as selectively expressed in the spleen, mesenteric lymph nodes, thymus, lung, NK cells, macrophages, and dendritic cells (13).

Interestingly, Myo1f knockout mice have also been reported to show increased susceptibility to infection by *Listeria monocytogenes* as a consequence of abnormally increased adhesion and reduced motility of neutrophils. This increased adhesion results from augmented exocytosis of β2 integrin-containing granules (14).

This study examines the capacity of 3BP2 to regulate Rho GTPase activity and mast cell migration and identifies Myo1f as a binding partner for 3BP2. Further, it characterizes Myo1f expression and distribution in mast cells and evaluates Myo1f function in adhesion, integrin expression, and SCF dependent migration in mast cells.

## Materials and Methods

### Cell Lines and Reagents

The LAD2 huMC line kindly provided by Drs. A. Kirshenbaum and D.D. Metcalfe (National Institutes of Health, Bethesda, MD) was grown in StemPro-34 media (Life Technologies, Carlsbad, CA), supplemented with StemPro-34 nutrient and L-glutamine (2 mM), penicillin (100 U/mL) and streptomycin (100 μg/mL), and 100 ng/mL SCF (Amgen, Thousand Oaks, CA) (15). The human mast cell line HMC-1 was obtained from J.H. Butterfield (Mayo Clinic, Rochester, MN, USA) and was grown in Iscove's medium supplemented with 10% heat-inactivated FBS, penicillin (100 U/ml), and streptomycin (100 μg/ml) (16). COS-7 cell line was cultured in Dulbecco's Modified Eagle Medium (DMEM), 10% FCS, 1% penicillin-streptomicin (mixture 5k/5k), 1% L-glutamine A 200 mM.

### Antibodies and Other Reagents

Mouse antibodies, α-3BP2 C5, α-3BP2 C11, α-Myo1f C5, α-KIT (clone Ab81), and rabbit α-Kit (H300) were purchased from Santa Cruz (Santa Cruz Biotechnology, Inc. Santa Cruz, CA). Mouse anti-CD29-APC (α-integrin β1) clone MAR4 from BD Pharmigen (BD Biosciences, San José, CA), mouse α-integrin β7-PE from Biolegend (San Diego, CA), goat α-mouse alexa-647, and goat α-rabbit alexa-488 were from Life Technologies (Carlsbad, CA), mouse α-human-FcεRI-PE from eBioscience (San Diego, CA). Mouse α-Rac1, α-RhoA, and α-Cdc42 antibodies were from Cytoskeleton (Cytoskeleton Inc., Denver, CO), mouse α-Rac2 antibody was from antibodies-online. Antiphosphotyrosine (pTyr) monoclonal was obtained from Zymed Laboratories (Invitrogen Life Technologies, Carlsbad, CA). Biotinylated human IgE (IgEB) was obtained from Abbiotec (San Diego, CA, USA). Anti-mouse peroxidase Ab was obtained from DAKO (Carpinteria, CA, USA). Streptavidin, the tyrosine kinase inhibitor sunitinib malate, puromycin, poly-lysine-D, fibronectin, doxycycline hyclate, mouse α-tubulin (DM1A), and mouse α-flag (m2Ab) were purchased from Sigma (Sigma-Aldrich, St. Louis, MO, USA). α-pKIT Tyr703 was from Cell Signaling (Cell Signaling Technology, Danvers, MA) and α-GPF from Roche (Roche Molecular Biochemical, Pleasanton, CA). Goat α-rabbit-HRP was from Life Technologies (Life Technologies).

### Cell Activation or Inhibition

Cells were starved overnight in culture media without SCF. The following day, cells were stimulated with 100 ng/ml of SCF in Tyrode's buffer for the indicated times. For IgE-dependent activation we sensitized cells with biotinylated IgE (0.1 μg/ml) overnight, and stimulated them for 30 min at 37°C with streptavidin (0.4 μg/ml) to induce IgE crosslinking. For inhibition, cells were incubated with Sunitinib for 30 min at 37°C in Tyrode's Buffer, DMSO was used as a control.

### Immunofluorescence Assays

Cells were activated or inhibited as described above. Afterwards, cells were fixed in PFA 4%—phosphate buffered saline (PBS) at 4°C. Then, cells were seeded on a poly-lysine-D coated plate with a Cytospin device (50.000 cells/sample). Cells were permeabilized with Saponin buffer (PBS-0.05% Saponin) for 15 min at 4°C. Afterwards, we used blocking buffer [0.2% skimmed milk, 2% FCS, 1% bovine serum albumin (BSA), 0.01% triton X-100, 0.01% NaN_3_, 20% Rabbit Serum (or 20% FCS), dissolved in PBS] for 1 h at 4°C. We used primary antibodies (0.1–0.2 μg/100.000 cell) for 2 h of incubation at 4°C. At last, we used goat anti-mouse or goat anti-rabbit secondary antibodies labeled with Alexa-488 or Alexa-647 for 45 min (dilution 1:300 – 1:500) at 4°C. For nuclei staining we used Hoechst stain (dilution 1:10.000 in PBS-0.1% BSA), and for actin staining we used phalloidin-TRITC (dilution 1:500 – 1:1000). Preparations were visualized with a Leica SP5 confocal microscope. All images were processed with Fiji/ImageJ free software (17).

### Yeast Three-Hybrid Assay

The yeast three-hybrid system was performed as described elsewhere (18). The 3BP2 adaptor cloned in the bicistronic pBridge vector (which carries the src-Fyn clone) and transformed in the yeast strain CG1945 was used as a bait to screen a human bone marrow matchmaker cDNA library cloned in pACT2 (Clontech Laboratories Inc, Mountain View, CA, USA). Cotransformed clones that grew under restrictive conditions were then tested using the β-galactosidase assay. The β-galactosidase liquid culture assay using *o*-nitrophenyl β-D-galactopyranoside as a substrate was carried out as described in the Clontech yeast protocols handbook. Positive clones were processed to purify their plasmids and they were sequenced.

### Active GTPase Assays

GTPase activation assays were performed using Rac1, Cdc42, and RhoA G-LISA kits following manufacturer's instructions (Cytoskeleton) as well as described elsewhere (19). Briefly, Cell lysates (5 × 10^5^ to 2 × 10^6^ cells) were prepared using G-LISA lysis buffer, snap-frozen in liquid nitrogen and processed within 2 weeks after preparation. Lysate aliquots, corresponding to Rac1-Rac2, Cdc42, and RhoA assays, were applied, respectively, to wells coated with Rac1, Cdc42, or Rho-GTP-binding protein. Active, GTP-bound GTPases bound to the wells were detected with the corresponding specific Ab. Constitutively active proteins were used as a standard.

### Immunoprecipitation, Immunoblotting, and COS Transfection

Cells were treated with Sunitinib as described above. Whole cell lysate preparations were obtained as described elsewhere (20). Immunoprecipitation experiments were conducted using a procedure described elsewhere (5). COS-7 cells were transiently transfected using Nucleofector (Cell Line Nucleofector Kit V from Lonza Cologne AG, Lonza) following the manufacturer's instructions. Cells were transfected with a flag-tagged Myo1f construct from (OriGene Technologies, Rockville, MD), cloned into a pCMV6-Entry plasmid. 3BP2 cloned in pCDNA 3.1 and Fyn cloned in psrα used in COS transfections were described in Saborit et al. (21) and Alvarez-Errico et al. (22), respectively. SH2-3BP2-GFP construct was obtained cloning the SH2 domain of 3BP2 into a pN3-EGFP construct (Clontech Laboratories, Inc, Mountain View, CA, USA); pN3-EGFP plasmid was used as control.

### Lentiviral Transduction

Lentiviral particles to silence the 3BP2 gene expression were generated using Mission shRNA technology, according to the manufacturer's instructions (Sigma-Aldrich, St. Louis, MO) as described elsewhere (5, 6). The lentivirus with shRNA sequences A, B, C, or D for Myo1f were from Origen Technologies. The TRCN0000158118 sequence that overlaps with sequence D in the Origen plasmids (CGTCTTCAAGACCGAGTTTGT) was furthered cloned into a Tet-pLKO-puro plasmid [Addgene plasmid 21915; (23)] which expression is inducible by doxycycline (0.5 μg/mL) and used for subsequent experiments.

The Lenti ORF clone of human SH3-domain binding protein 2 (SH3BP2), transcript variant 1, mGFP tagged and Lenti vector with C-terminal monomeric GFP tag as a control (OriGene Technologies) were used for 3BP2 overexpression experiments.

### Migration Assays

Migration assays were performed using transwell polycarbonate membranes from Costar (8 μm pores, Corning Incorporated, Kennebunk, ME) suitable for mast cells as reported in Kataoka et al. (24). Briefly, LAD2 cells were starved O/N in complete media without SCF and, when necessary, with biotinylated IgE. The next day, 1 × 10^5^ cells per point were washed with fresh StemPro-34 media and dropped into the upper transwells. After 10 min for stabilization we added the stimulus on the bottom well (100 ng/mL of SCF, 0.4 μg/ml streptavidin or StemPro Supplement 1x), and the cells were left to migrate for 4 h at 37°C and 5% CO_2_. After 4 h the cells were rinsed with an EDTA-NaCl-PBS buffer to detach them from the bottom of the upper chamber, they were stained with crystal violet and counted under optic microscopy. In all cases, the cells in the upper chamber were counted after 4 h with Trypan blue to evaluate viability.

### Adhesion Assays

P96 plates were coated O/N with fibronectin (20 μg/mL), poly-lysine-D (0.1% w/v) or PBS. Next day, wells were washed with PBS, blocked with 5% BSA and washed again with PBS. 1–5 × 10^4^ cells starved O/N were dropped in triplicate wells and activated with 100 ng/mL SCF in complement-free StemPro-34 media for 30 min. After that, wells were rinsed 3 times with PBS and the number of remaining cells was assessed with CellTiter-Glo® Luminescent Cell Viability Assay from Promega (Promega Corporation, Madison, WI).

### FACS Staining

β7 and β1 integrins, KIT and FcεRI expression were detected by direct staining with the indicated Abs for 30 min at 4°C. Cells were then analyzed using a FACSCalibur flow cytometer (FACScan; BD Biosciences). In all cases dead cells were excluded based on their Forward (FSC) and side scattering (SSC) profile.

### Data Analysis

All results are expressed as mean ± standard error of the mean (SEM). Unpaired student's *t*-test or one-way ANOVA were used to determine significant differences (*p*-value) between two or several experimental groups, respectively, after determination of normal distribution of the sample and variance analysis.

## Results

### 3BP2 Is Critical for SCF-Dependent Mast Cell Migration

**Figure 1:**
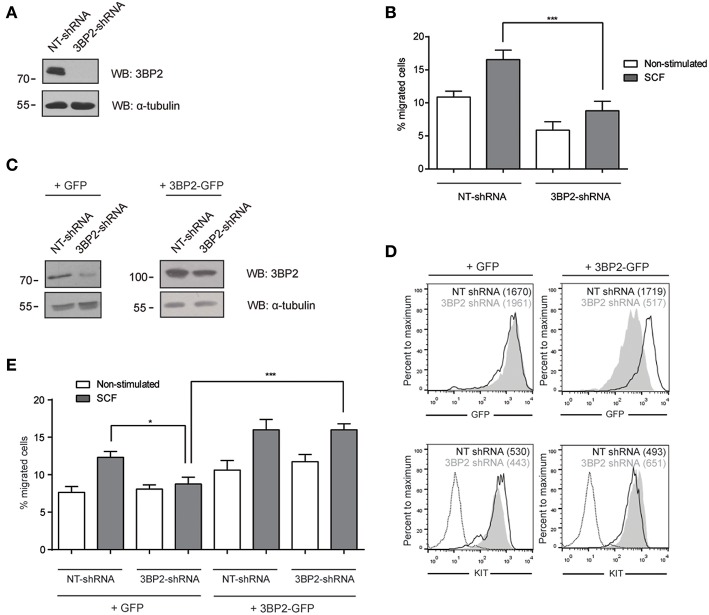
3BP2 knockdown impairs SCF-dependent mast cell migration. **(A)** Western blot showing the 3BP2 shRNA knockdown in LAD2 cells. **(B)** Percentage of migrated non-target control (NT-shRNA) o 3BP2-knockdown (3BP2-shRNA) LAD2 mast cells toward SCF gradient (100 ng/mL). **(C)** Western blot determination of the reconstitution of both groups with 3BP2-GFP or GFP alone. **(D)** GFP and KIT receptor expression determined by flow cytometry. NT-shRNA is represented by an empty black curve and 3BP2-shRNA is represented by a filled gray curve. Mean intensity of fluorescence (MIF) of each group is represented in parentheses. **(E)** Percentage of cell migration after 4 h chemotaxis with SCF (100 ng/mL) of silenced and reconstituted cells, as indicated in the figure. Viability was tested in the upper wells after the assay. The one-way ANOVA test was used for the statistical analysis (*p < 0.05, ***p < 0.001). Migration data are the mean of three independent experiments.

Given that 3BP2 is required for proper KIT signaling (6), and SCF, the ligand for KIT, is a major chemotactic factor in mast cells (1), this study explores the capacity of 3BP2 to modulate SCF-dependent chemokinesis. Migration assays were carried out using Transwell chambers, with SCF as the stimuli. 3BP2 shRNAs sequences were validated in previous works (5, 6). Our data show that 3BP2-silenced LAD2 cells (Figure 1A) do not migrate toward an SCF gradient (Figure 1B). As described previously, 3BP2 knockdown cells downregulate KIT expression, thereby leading to cell apoptosis (6). The migration experiments were performed under the same conditions in which cell survival and KIT expression were not significantly affected (first week of transduction). The viability was tested in all these conditions to rule out a lack of migration due to cell apoptosis (data not shown). To ensure the specificity of this event, silenced cells were reconstituted for 3BP2 using a plasmid-encoding 3BP2-GFP protein or a control GFP protein (Figure 1C). The GFP and KIT expression levels were monitored to ensure good transfection efficiency before the migration tests were carried out (Figure 1D). As expected, 3BP2 reconstitution reestablished the capacity for cell migration (Figure 1E).

**Figure 2:**
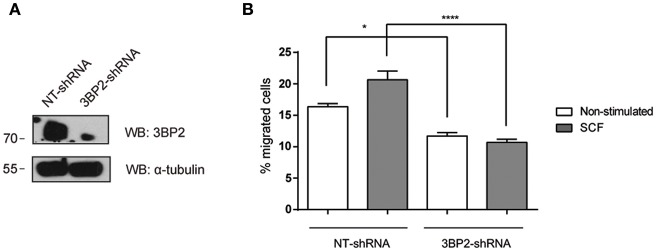
3BP2 knockdown decreases migration of mast cells harboring the KITD816V mutation. **(A)** Western blot showing the 3BP2 shRNAs knockdown in HMC-1 cells. **(B)** Percentage of migrated HMC-1 mast cells silenced for 3BP2 (3BP2-shRNA) or non-target control (NT-shRNA) toward an SCF gradient (100 ng/mL). The student's *t*-test was used for the statistical analysis (*p < 0.05, ****p < 0.0001). In all migration assays, viability was tested in the upper wells after the assay. Data are the mean of three independent experiments.

Next, we analyzed whether 3BP2 was also needed for mutant KIT D816V-mediated migration. KIT D816V is a hallmark of mastocytosis, a rare disease caused by the accumulation of functionally defective mast cells. For that we used HMC-1 cell line in which KIT has the gain-of-function mutation D816V. As shown in Figure 2, 3BP2 silencing impairs cell migration toward SCF in HMC-1 cells. In this case migration basal levels are higher than those observed in LAD2 mast cells since KIT signaling is constitutively active. Although the mutation confers receptor activation in absence of SCF, SCF addition still increases cell activation as reported by us and others (6, 25). Altogether our data indicates that 3BP2 provides signals needed for SCF-dependent mast cell migration.

### 3BP2 Is Essential for FcεRI-Mediated Mast Cell Migration

**Figure 3:**
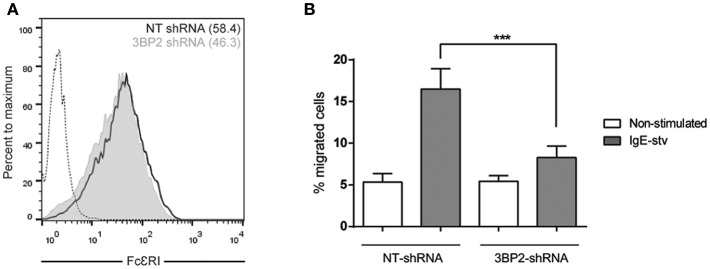
3BP2 also reduces significantly IgE-dependent cell migration. **(A)** FcεRI expression in NT and 3BP2-silenced LAD2 cells. **(B)** Percentage of cell migration after 4 h chemotaxis with IgE+ Streptavidin in NT and 3BP2-silenced cells. Viability was tested in the upper wells after the assay. The one-way ANOVA test was used for the statistical analysis (***p < 0.001). Migration data are the mean of three independent experiments.

It has been reported that Lyn and Syk are essential for FcεRI-mediated migration (26). Since the adaptor molecule 3BP2 coprecipitates with Lyn and Syk in mast cells and 3BP2 silencing leads to a decrease in Syk phosphorylation (5) we next assessed the ability of 3BP2 to promote FcεRI-mediated migration. For that purpose non target or 3BP2 silenced LAD2 cells were sensitized with biotinylated IgE overnight. Migration assays were performed using streptavidin as stimuli (mimicking the allergen). As shown in Figure 3, 3BP2 silencing leads to an impairment in migration toward the streptavidin. Levels of FcεRI in both types of cells were similar. As reported previously, 3BP2 silencing does not affect FcεRI expression levels (5). To check whether 3BP2 role in chemotaxis goes beyond FcεRI or KIT receptors we carried out migration assays using the StemPro 34 supplement as stimuli. This supplement contains a wide array of growth factors needed for growth and proliferation of LAD2 cells. Interestingly, cells where 3BP2 was silenced show less migration toward the stimuli suggesting an overall role of 3BP2 in LAD2 migration (Figure S1).

### The 3BP2 Adaptor Protein Is Required for Rac2 and Cdc42 Activation in Mast Cells

**Figure 4:**
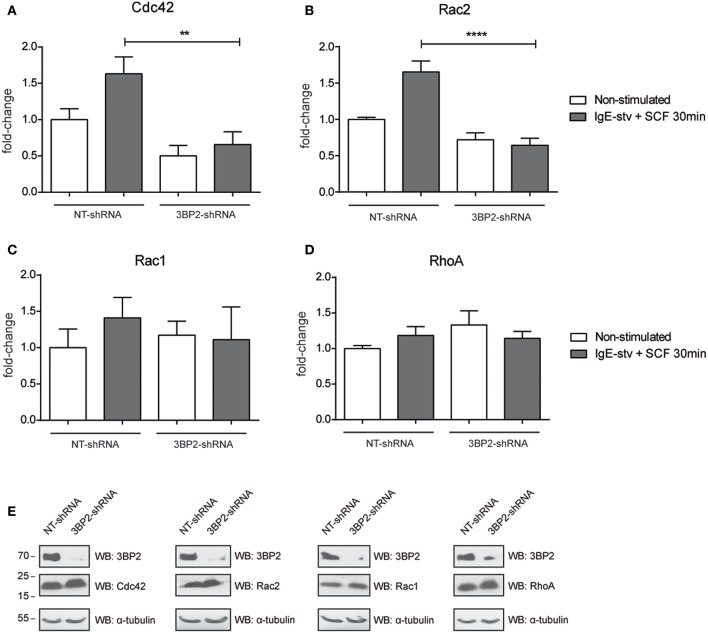
3BP2 knockdown impairs Cdc42 and Rac2 activation in mast cells. G-Lisa quantification of GTP-bound (active) small GTPases Rho **(A)** Cdc42; **(B)** Rac2, **(C)** Rac1, and **(D)** RhoA were performed before and after activation by IgE-stv plus SCF for 30 min in LAD2 cells. **(E)** Western blot showing GTPases levels in NT and 3BP2-silenced cells. The one-way ANOVA test was used for the statistical analysis (**p < 0.01, ****p < 0.0001). Data are the mean of three independent experiments.

The adaptor molecule 3BP2 has been proposed as a key molecule for Rho family GTPase activation and migration in neutrophils (7). The Rho family of GTPases undergoes activation by GTP/GDP exchange and controls cytoskeletal rearrangements (9). First we characterized the activation pattern of the Rho GTPase family in the human mast cell line LAD2 (Figure S2). Activation of Rac1, Rac2, and Cdc42 increases after IgE and SCF stimulation. Thus, we then performed an assay to determine RhoA/Rac1/Rac2/Cdc42 activity in 3BP2-silenced LAD2. Our results show that Cdc42 and Rac2 activation was significantly impaired in 3BP2-silenced cells after activation. Interestingly, Rac-1 was not modified, thus indicating that 3BP2 is selectively relevant for activation of some members of Rho family GTPases (Figure 4). The data suggest the involvement of 3BP2 in pathways associated with cell motility and actin dynamics.

### Myo1f as a Novel Ligand for 3BP2

**Figure 5:**
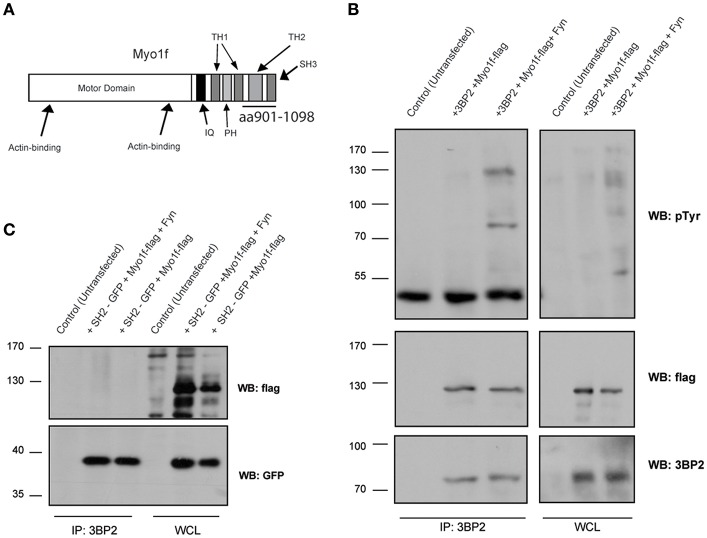
Myo1f, a new ligand for 3BP2. **(A)** Representation of Myo1f. The clone identified through the three-hybrid assay was the SH3 region of this protein (aa901-1098). **(B)** Immunoprecipitation of the 3BP2 protein in COS-7 cells transfected with 3BP2 and Myo1f-flag, with or without the presence of Fyn kinase. Membrane was blotted with α-pTyr, α-flag, and α-3BP2 antibodies. **(C)** Immunoprecipitation of 3BP2 in COS-7 cells transfected with the SH2 domain of 3BP2 (SH2-GFP) and Myo1f-flag, with or without the presence of Fyn kinase. Membrane was blotted with α-flag and α-GFP antibodies.

In parallel to these studies, we screened a human bone marrow library using the yeast three-hybrid system used previously (18, 21), this time with 3BP2 as the bait in order to search for new ligands for 3BP2. Our screening process rendered known partners for 3BP2, including Vav1 (data not shown) and Myo1f, a novel ligand. Myo1f is a long-tailed class I myosin that consists of an N-terminal motor domain, light-chain-binding IQ motifs, a basic tail homology 1, a proline-rich TH2 domain and a TH3 domain containing a single Src homology 3 (SH3) domain. A positive clone observed in the screening process encoded the c-terminal region of the protein containing the complete SH3 domain (Figure 5A). Moreover, 3BP2 coprecipitated with Myo1f in transfected COS cells in the absence or presence of Fyn kinase (Figure 5B), thereby indicating an interaction that is independent of phosphorylation. Interestingly, Myo1f and 3BP2 are susceptible to phosphorylation by Fyn kinase, thus suggesting regulation of these proteins upon phosphorylation. In order to check whether Myo1f can also be recruited by the SH2 domain of 3BP2, we performed an immunoprecipitation experiment in COS cells transfected with only the SH2 domain of 3BP2 in the presence of Fyn. Our findings revealed that no binding was observed in these conditions (Figure 5C).

### Myo1f Is Expressed in Mast Cells and Colocalizes With 3BP2

**Figure 6:**
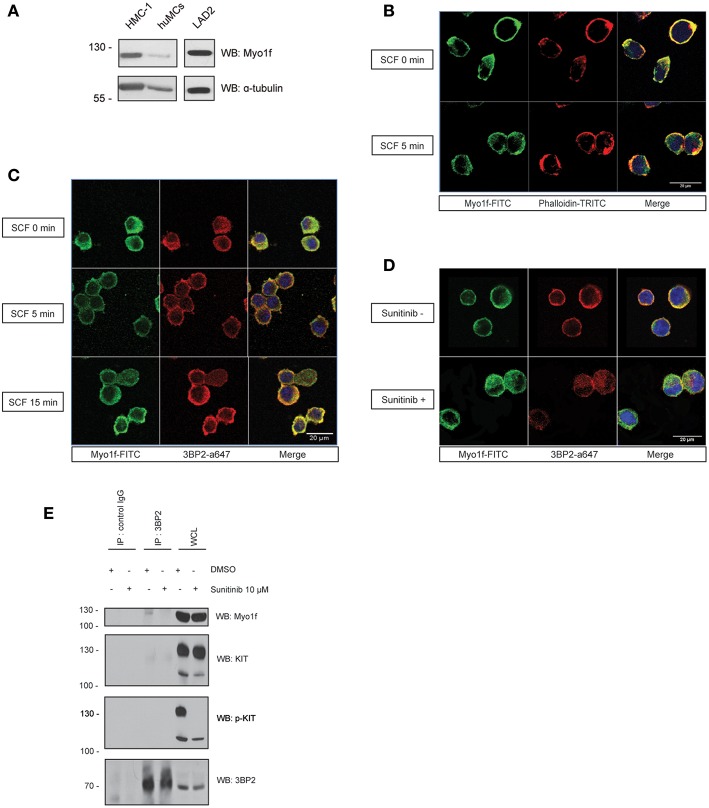
3BP2 and Myo1f colocalization is modulated by KIT signaling in mast cells. **(A)** Expression of Myo1f in the mast cell lines HMC-1 and LAD2 and in CD34+-derived human mast cells (huMCs). **(B)** Immunofluorescence of LAD2 mast cells showing Myo1f (green), actin staining (phalloidin; red) and the nucleus (Hoechst; blue) after 5 min of stimulation with SCF (100 ng/mL) or resting (0 min). **(C)** Immunofluorescence of LAD2 mast cells showing Myo1f (green), 3BP2 (red) and the nucleus (Hoechst; blue) after stimulation for different times with SCF (100 ng/mL) or resting (0 min). **(D)** Immunofluorescence of HMC-1 cells treated with sunitinib (10 μM) or untreated showing Myo1f (green), 3BP2 (red) and the nucleus (Hoechst; blue). **(E)** Immunoprecipitation of 3BP2 in HMC-1 cells treated with sunitinib (10 μM) or control (DMSO). Membrane was blotted with α-Myo1f, α-3BP2, and α-KIT/α-pKIT antibodies to check KIT inhibition by sunitinib.

Next, we determined the expression of Myo1f in human mast cells. Mast cell lines LAD2 and HMC-1 and CD34^+^-derived human mast cells express Myo1f, as confirmed by western blot in our study (Figure 6A). Myo1f colocalizes with the cortical actin network in resting conditions and after SCF stimulation in LAD2 cells (Figure 6B). Myo1f and 3BP2 showed some localization under resting and SCF-activated conditions. Interestingly, Myo1f and 3BP2 colocalization was found mostly on the cell membrane at 5 min (Figure 6C). Our data suggest that KIT activation may modulate the 3BP2-Myo1f interaction. In that context, in HMC-1 cell line, where KIT is constitutively active, 3BP2 and Myo1f colocalized at the plasma membrane. Remarkably, the KIT kinase inhibition with sunitinib altered the colocalization (Figure 6D) and 3BP2-Myo1f coprecipitation is abrogated in those conditions (Figure 6E). These data indicate that KIT signaling can regulate the Myo1f-3BP2 interaction.

### Myo1f Knockdown Does Not Impair SCF-Dependent Mast Cell Migration

**Figure 7:**
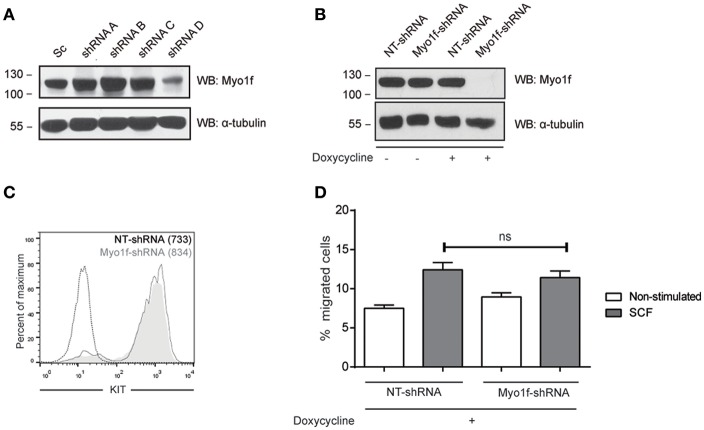
Myo1f knockdown does not affect SCF-dependent migration. **(A)** Western blot showing the Myo1f shRNAs tested. **(B)** Western blot showing Myo1f knockdown and **(C)** membrane surface expression of KIT receptor in LAD2 mast cells silenced for Myo1f (Myo1f-shRNA) or non-target control (NT-shRNA) after doxycycline induction (0.5 μg/mL). **(D)** Percentage of migrated LAD2 mast cells silenced for Myo1f (Myo1f-shRNA) or non-target control (NT-shRNA) toward an SCF gradient (100 ng/mL) with doxycycline induction (0.5 μg/mL). The student's *t*-test was used for the statistical analysis. In all migration assays, viability was tested in the upper wells after the assay. Data are the mean of three independent experiments.

We next assessed whether Myo1f knockdown can modulate KIT functions by analyzing cell migration. Various Myo1f shRNA sequences were tested in LAD2 cells (Figure 7A). Only one shRNA sequence showed significant Myo1f silencing and was further cloned under doxycycline-inducible plasmid to control Myo1f silencing and reconstitution at will. Western blot was performed to corroborate the knockdown data (Figure 7B), and KIT expression levels were evaluated by flow cytometry in non-target cells vs. Myo1f-silenced cells (Figure 7C). Our results show that Myo1f knockdown does not affect the capacity of mast cells to migrate toward SCF under these conditions (Figure 7D). Viability was also monitored after migration and showed no changes (data not shown).

### β1 (CD29) and β7 Integrin Expression Are Affected in Myo1f and 3BP2 Silencing

**Figure 8:**
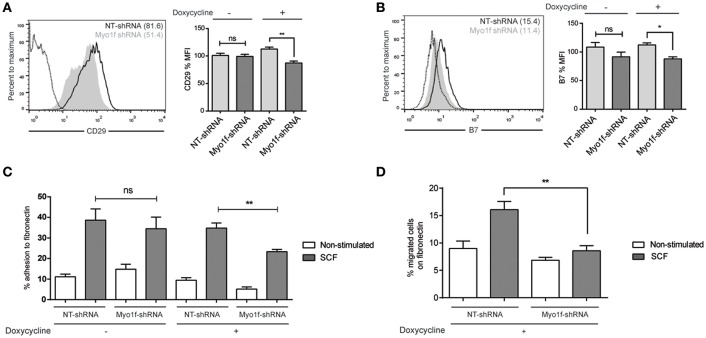
β1 and β7 integrin expression are reduced as well as adhesion and migration to fibronectin in Myo1f knockdown mast cells. **(A)** Flow cytometry analysis of β1 (CD29) expression on NT and Myo1f shRNA LAD2 cells, **(B)** β7 integrin expression in NT shRNA and Myo1f shRNA. NT shRNA is represented by an empty black curve and Myo1f shRNA is represented by a filled gray curve. The mean of each group is represented in parentheses. **(C)** NT shRNA cells and Myo1f shRNA LAD2 cells were assayed for adhesion in fibronectin-coated wells, stimulated with SCF (100 ng/mL) for 30 min, and the percentage of adhesion was determined. **(D)** NT shRNA and Myo1f shRNA LAD2 mast cells were assessed for migration for 4 h in fibronectin-coated Transwell chambers and then counted under optic microscopy. Viability was tested in the upper wells after the assay. The student's *t*-test **(A,B)** or ordinary one-way ANOVA test **(C,D)** was used for the statistical analysis (*p < 0.05, **p < 0.01). The bar chart data correspond to the mean of at least three independent experiments.

It has been reported that neutrophils from Myo1f-deficient mice exhibit abnormally increased adhesion and reduced motility as a result of augmented exocytosis of β2-integrin–containing granules and a reduction in cortical actin (14). β1 and β7 integrins are responsible for migration and homing in SCF-dependent mast cell migration (27). Thus, we analyzed the integrin profile of β1 (CD29), β2 (CD18), and β7 chains in Myo1f vs. control cells. β2 expression was very low in mast cells under various treatments, and no differences were observed in silenced or control cells (data not shown). Conversely, β1 and β7 integrins were well expressed in mast cells, and no significant differences were observed between untreated and SCF-stimulated cells in terms of membrane expression measured by flow cytometry (data not shown). Interestingly, Myo1f-silenced cells were found to express significantly less β1 integrin, in comparison with non-target cells (Figure 8A). Our results also show mild but significant differences in β7 expression between control cells and Myo1f-silenced cells (Figure 8B).

SCF-dependent mast cell migration is preceded by an increase in cell adhesion, a process that is dependent on integrins (28). Next we assessed whether the differential expression of integrins on Myo1f silencing affects SCF-dependent cell adhesion. As shown in Figure 8C, Myo1f silencing induced by doxycycline affected SCF-induced adhesion to fibronectin.

Finally, we measured the capacity of these cells to migrate toward an SCF chemotactic gradient through a fibronectin-coated surface. Our data show that Myo1f-silenced cells had a significantly lower migration capacity under these conditions, thus indicating that this impaired adhesion affected mast cell migration (Figure 8D).

**Figure 9:**
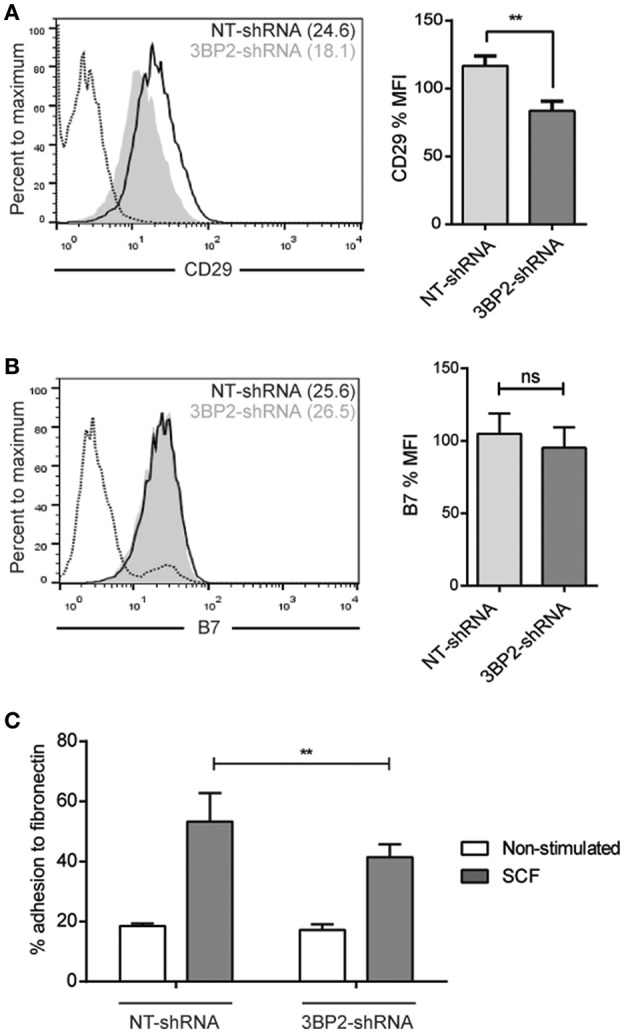
3BP2 silencing decreases β1 expression and significantly impairs adhesion to fibronectin. **(A)** Flow cytometry analysis of β1 (CD29) expression on NT and 3BP2 shRNA LAD2 cells, **(B)** β7 integrin expression in NT shRNA and 3BP2 shRNA. NT shRNA is represented by an empty black curve and 3BP2 shRNA is represented by a filled gray curve. The mean of each group is represented in parentheses. **(C)** NT shRNA cells and 3BP2 shRNA LAD2 cells were assayed for adhesion in fibronectin-coated wells, stimulated with SCF (100 ng/mL) for 30 min, and the percentage of adhesion was determined. The student's *t*-test **(A,B)** or ordinary one-way ANOVA test **(C)** was used for the statistical analysis (**p < 0.01). The bar chart data correspond to the mean of at least three independent experiments.

We conducted similar experiments in 3BP2 silencing cells assessing integrins expression profile. We found that β1 integrin expression was significantly reduced but β7 integrin expression was unaffected in 3BP2-silenced cells (Figures 9A,B). We found as well significant differences in cell adhesion to fibronectin in 3BP2-silenced cells (Figure 9C). These differences may also contribute to the capability of cells to migrate. As we show above when 3BP2 expression is low or almost absent cell migration is more grossly affected that the one observed in Myo1f-silenced cells.

## Discussion

Migration of mast cells is required for their recruitment to target tissues and for their infiltration to inflammation sites associated with chronic atopic diseases, or during bacterial or parasitic infection. Thus, mast cells recognize chemotactic stimuli and trigger a signaling cascade of events that lead to integrin activation, adhesion and migration. SCF, the KIT ligand, is a key chemotactic factor and is crucial for mast cell proliferation, survival, homing and migration. Proper KIT signaling is required to ensure that these events progress properly. Some years ago, our group showed that the 3BP2 adaptor protein previously described as critical for mast cell degranulation through FcεRI (5) also regulates survival through KIT receptor expression and signaling (6). As we reported, 3BP2 regulates KIT transcription through microphthalmia-associated transcription factor (MITF), described to control KIT expression. Thus, reduced 3BP2 expression is associated with decreased KIT expression and increased cellular apoptosis (6).

In this study we show that 3BP2 also plays a role in SCF-dependent cell migration. Since the silencing of 3BP2 ends up reducing KIT expression and compromising cell viability, all experiments we performed were monitored for KIT expression and cell viability. The mechanisms underlying 3BP2-modulated cell migration may be related to its role as a scaffold protein, since with its binding partners it builds a molecular network that contributes to the KIT signalosome. Indeed, the silencing of 3BP2 reduces PI3K and MAP kinase signaling after SCF engagement (6). This work used the yeast three-hybrid system to screen a human bone marrow library, using human 3BP2 as bait, and identified Vav1 (clone encoding the SH2 domain; amino acids 665-754) and Myo1f (clone encoding SH3 domain; amino acids 901-1185) as ligands for 3BP2. Vav1 has been reported to bind to 3BP2 in a phosphorylation-dependent manner, thus increasing NK cell killing (10, 21, 29) and B cell activation (30). 3BP2 has been reported as crucial for full activation of Vav1 in neutrophils, thereby supporting a role for the adaptor in neutrophil activation and migration through activation of Rho family of GTPases (7). Rho GTPases, specifically Rac1,2,3, RhoA and Cdc42, have been shown to regulate various exocytosis-related events, including actin remodeling, exocytic complex formation and calcium influx, across a number of cell models (31). Although their exact mechanism of regulation may vary between cells, it appears that Rho GTPases are predominantly responsible for regulating actin remodeling. Rac1 and Cdc42 have been reported to stimulate mast cell exocytosis, while RhoA has no effect (32). Although Rac1 and Rac2 are 92% identical, they have been shown to have distinct cellular functions (33). Therefore, a lack of Rac2 results in diminished chemotaxis and degranulation in mast cells, thus defining distinct functions for Rac2 that cannot be compensated for by Rac1 (34, 35). This is consistent with previous data where we reported that 3BP2 silencing expression inhibits mast cell degranulation (5) and with the present data, which showed that Rac2 and Cdc42 activation was impaired in 3BP2 knockdown mast cells in which chemotaxis was diminished. Interestingly, Rac2 knockout exhibits a reduction in mast cell survival (34) as occurs in 3BP2 silenced cells (6), thus suggesting that both molecules are involved in a common signal pathway.

The mechanism used for 3BP2 to connect KIT triggering with small GTPase activity has not been elucidated. 3BP2 is known to coprecipitate with KIT in mast cells but no direct binding to the receptor has been reported (6). Src kinases Lyn, Fyn and PI3K are ligands for KIT binding to Y568, Y570, and Y721, respectively, in the cytoplasmic tail of the human receptor. On the other hand, 3BP2 can be phosphorylated by Fyn and Lyn (30, 36) and has been reported to be associated with Src kinases (5, 36) and PI3K (29). It is therefore conceivable that KIT and 3BP2 are linked through these common partners. Interestingly, Fyn-dependent axis on KIT activation leads to the phosphorylation of Gab2 and cytoskeleton reorganization and mast cell migration through Rac GTPase activation (37–40). Studies with different murine KIT mutants showed the importance of Y567 and Y719 for KIT–mediated chemotaxis. Phosphorylated Y567 recruits Src kinases Lyn or Fyn, and this results in activation of the p38 pathway, which is also important for chemotaxis (39, 41). Interestingly, 3BP2 silencing impairs the p38 pathway in mast cells after FcεRI triggering (5). Altogether, 3BP2 could act synergistically with Scr kinases, thus increasing the Rho family of small GTPases and chemotaxis. In contrast, phosphorylated Y179 recruits PI3K and the mediated and enhanced calcium signaling that has been found to be critical for chemotaxis (41). 3BP2 silencing has been reported to impair calcium signaling in mast cells (5). In summary, 3BP2, through common ligands, may modulate SCF-dependent MAP kinase activity and calcium influx that leads to activity of the Rho family of small GTPases, which is important for mast cell chemotaxis.

In this work, we identified Myo1f as a novel binding partner for 3BP2. Myo1f is an unconventional long-tailed myosin not involved in muscle contraction and whose expression is restricted to the immune system. However, its expression and role in mast cell function remains unclear. We showed that Myo1f is expressed in CD34-derived mast cells and mast cell lines, and colocalizes with the cortical actin ring. 3BP2-Myo1f interaction is independent of phosphorylation and possibly involves the SH3 domain of Myo1f and the proline-rich domain of 3BP2. However, it may be modulated after KIT activation. After 5 min of SCF stimulation, 3BP2 translocates to the membrane, thereby increasing its colocalization with Myo1f in that area. Myo1f membrane location is also more apparent after KIT engagement. This is consistent with an increase in PI3K activity upon KIT activation and production of phosphatidylinositol-3, 4, 5-triphosphate (PIP3), a binding site for PH-domain containing proteins like 3BP2 and Myo1f. In the HMC-1 mast cell line (harboring KITD816V), where KIT is constitutively active, colocalization of both proteins can be observed in the membrane area and by coprecipitation. Interestingly, coprecipitation is abolished in the presence of the kinase inhibitor sunitinib, that causes both molecules to distribute mostly in the cytoplasm. Overall, we concluded that Myo1f links 3BP2 to actin cytoskeleton after KIT stimulation.

Myo1f has been reported as key for neutrophil migration. Indeed, Myo1f knockout mice showed increased susceptibility to infection by *Listeria monocytogenes* due to an improper neutrophil migration to the infection sites (14). The authors reported that Myo1f prevents excessive exocytosis of β2 integrin-containing vesicles by regulating cortical F-actin or by controlling the final step of exocytosis (14). This type of integrin is dominant in integrin-mediated adhesion to the vascular endothelium, a process that is crucial for neutrophil migration to infected tissues (42, 43). Lately, Salvermoser and collaborators demonstrated that Myo1f is crucial for the dynamics of the deformation of the neutrophil nucleus and consequent capacity of these cells to extravasate *in vivo* rather than for integrin regulation (44). More recently, it has been shown that Myo1f regulates integrin-αVβ3 inducing M1-polarization in macrophages through PI3K/Akt/STAT signaling (45).

In our study, we explore the expression of several integrins after Myo1f knockdown. Our data showed that Myo1f knockdown decrease the cell surface expression of two integrin β chains, β1 (CD29) and β7, which are critical for mast cell adhesion and migration, usually coupled with the α4 chain. It has also been determined that Fyn kinase plays a role in mediating KIT/β1 integrin crosstalk in mast cells through Rac activation to promote spreading, cytoskeletal remodeling and migration (39, 40). Integrin α-4/β-1 (VLA-4) and α-4/β-7 are receptors for fibronectin (46). Consequently, we found that Myo1f-silenced cells have significantly reduced mast cell adhesion to fibronectin upon SCF activation. Moreover, migration of Myo1f-silenced mast cells was impaired, specifically on the integrin ligand (fibronectin)-coated surface, which resembles the physiological environment. Our results suggest that Myo1f may regulate integrin trafficking in mast cells and thus possibly affects integrin-vesicle exocytosis. Interestingly, we found that 3BP2 can also regulate β1 expression, thus indicating that both molecules are involved in that pathway, but cannot regulate β7 integrins, thereby showing a selective cascade for both molecules. β2 integrin levels in LAD2 were almost undetectable (data not shown), in line with the very low levels of β2 previously reported in human mast cells (47). We cannot discard that Myo1f may be acting in the integrin functionality as well. Integrins and actin are coupled through a physical linkage, which provides traction for migration (48). In that context, Myo1f would provide a link with the actin cytoskeleton and the adaptor protein 3BP2 delivering signals for adhesion to fibronectin and further cell migration. More experiments are needed to define the mechanism.

With respect to the role of Myo1f in the immune system, the Myo1f gene has been found to be fused to the mixed lineage leukemia (MLL) gene in acute monocytic leukemia (49). The presence of the Vav1-Myo1f fusion protein (where the C-terminal SH3 domain of Vav1 is replaced by the SH3 of Myo1f) was recently reported in peripheral T-cell lymphomas (PTLC), a heterogeneous group of non-Hodgkin lymphomas frequently associated with poor prognosis. This supports a possible role for the Myo1f SH3 domain in promoting the activity of the Vav1-Myo1f oncoprotein (50, 51). Furthermore, Myo1f has been proposed as a candidate gene for nonsyndromic deafness (52).

In summary, we have defined a key role for 3BP2 in SCF-dependent mast cell migration and identified a new partner, Myo1f, which links the adaptor to the actin cytoskeleton following KIT activation. This myosin regulates integrin-dependent adhesion and migration.

Further investigation is needed to clarify the mechanisms of Myo1f in the regulation of migration. The targeting of Myo1f could serve as a tool to help shed light on cytoskeleton changes to modulate mast cell function.

## Author Contributions

MM conceived the experiments, provided secure funding, wrote, and reviewed the manuscript. AN-F performed, conceived experiments, and reviewed the manuscript. EA-E performed experiments and reviewed the manuscript. ES-C provided technical support and reviewed the manuscript. JS conceived experiments and reviewed the manuscript.

### Conflict of Interest Statement

The authors declare that the research was conducted in the absence of any commercial or financial relationships that could be construed as a potential conflict of interest.
