# Supplementary figures and images for: Myo1f, an Unconventional Long-Tailed Myosin, Is a New Partner for the Adaptor 3BP2 Involved in Mast Cell Migration
Source: Front Immunol. 2019 May 9;10:1058. doi: 10.3389/fimmu.2019.01058 (PMC6521229; doi:10.3389/fimmu.2019.01058)

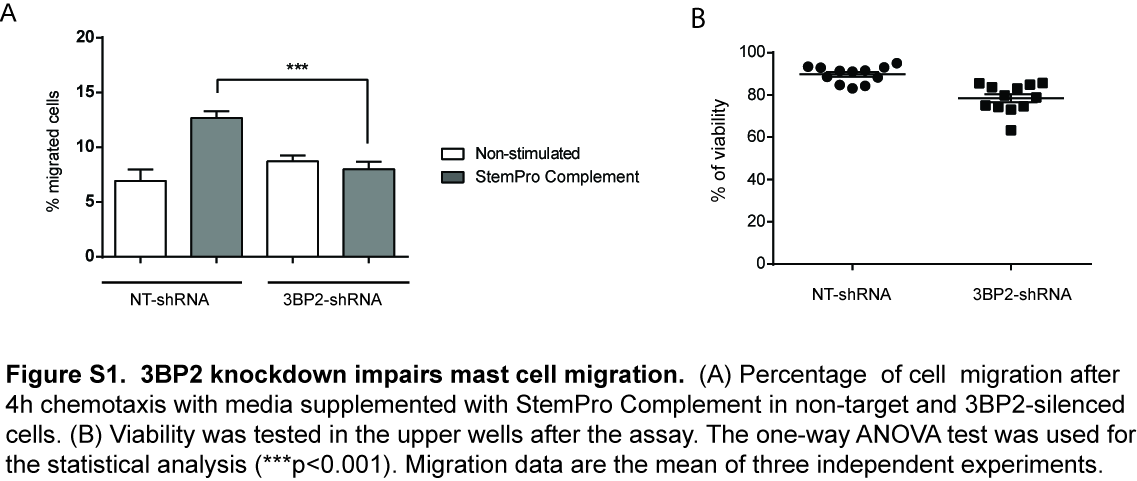

Supplement: Supplementary file 1 [file Image_1.TIF]

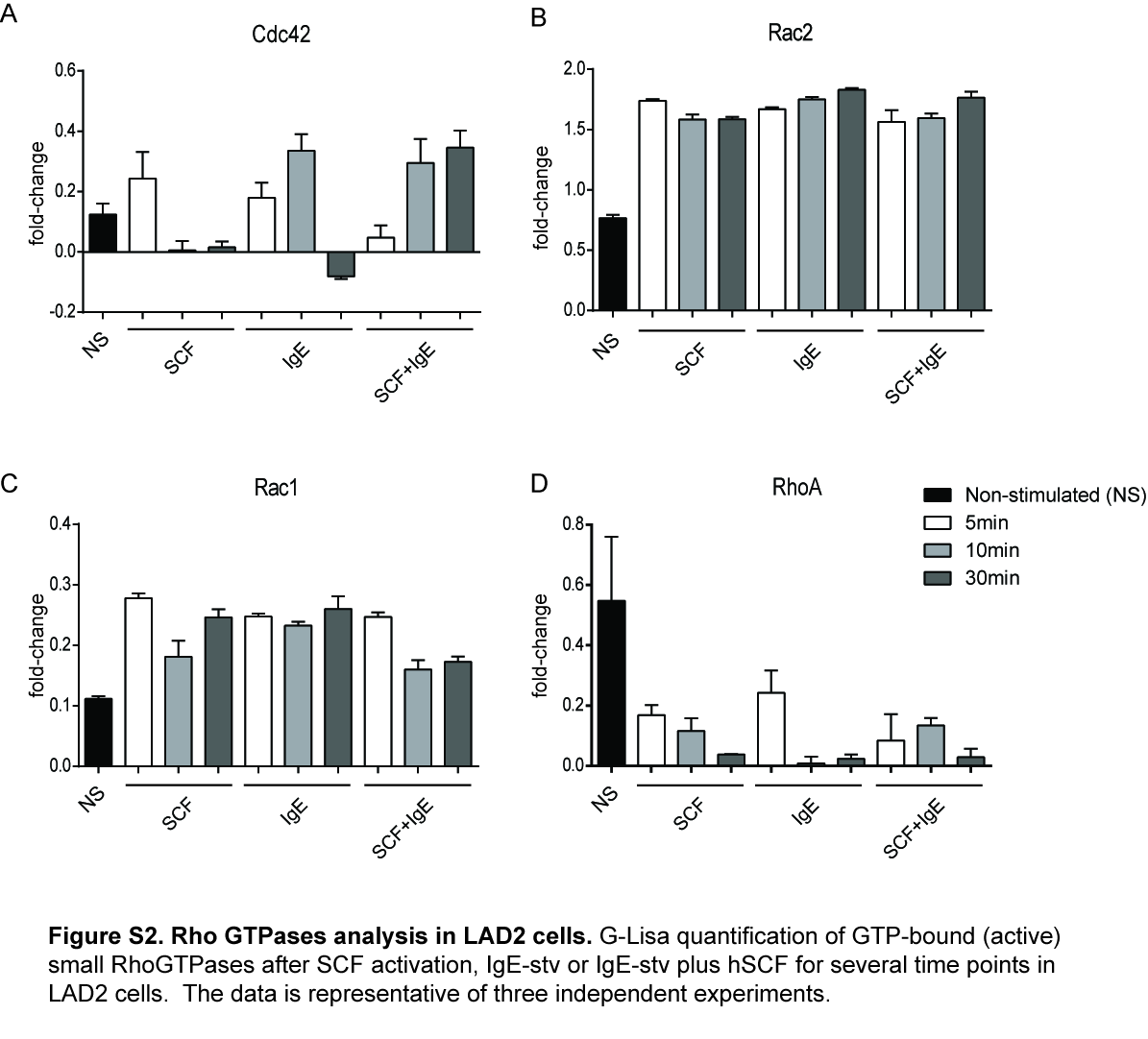

Supplement: Supplementary file 2 [file Image_2.TIF]
